# Non-driver mutations in myeloproliferative neoplasm-associated myelofibrosis

**DOI:** 10.1186/s13045-017-0472-5

**Published:** 2017-05-02

**Authors:** Bing Li, Robert Peter Gale, Zefeng Xu, Tiejun Qin, Zhen Song, Peihong Zhang, Jie Bai, Lei Zhang, Yue Zhang, Jinqin Liu, Gang Huang, Zhijian Xiao

**Affiliations:** 1 MDS and MPN Centre, Institute of Hematology and Blood Diseases Hospital, Chinese Academy of Medical Sciences & Peking Union Medical College, 288 Nanjing Road, Tianjin, 300020 China; 2 State Key Laboratory of Experimental Hematology, Institute of Hematology and Blood Diseases Hospital, Chinese Academy of Medical Sciences & Peking Union Medical College, Tianjin, China; 3 Haematology Research Centre, Division of Experimental Medicine, Department of Medicine, Imperial College London, London, UK; 4 Medical Service Division, Institute of Hematology and Blood Diseases Hospital, Chinese Academy of Medical Sciences & Peking Union Medical College, Tianjin, China; 5 Department of Pathology, Institute of Hematology and Blood Diseases Hospital, Chinese Academy of Medical Sciences & Peking Union Medical College, Tianjin, China; 6 Divisions of Experimental Hematology and Cancer Biology, Cincinnati Children’s Hospital Medical Center, Cincinnati, OH USA

**Keywords:** Myeloproliferative neoplasm-associated myelofibrosis, Non-driver mutation, Targeted gene sequencing

## Abstract

We studied non-driver mutations in 62 subjects with myeloproliferative neoplasm (MPN)-associated myelofibrosis upon diagnosis, including 45 subjects with primary myelofibrosis (PMF) and 17 with post-polycythemia *vera* or post-essential thrombocythemia myelofibrosis (post-PV/ET MF). Fifty-eight subjects had ≥1 non-driver mutation upon diagnosis. Mutations in mRNA splicing genes, especially in *U2AF1*, were significantly more frequent in PMF than in post-PV/ET MF (33 vs. 6%; *P* = 0.015). There were also striking differences in clonal architecture. These data indicate different genomic spectrums between PMF and post-PV/ET MF.

**Electronic supplementary material:**

The online version of this article (doi:10.1186/s13045-017-0472-5) contains supplementary material, which is available to authorized users.

## To the Editor

MPN-associated myelofibrosis is a clinical descriptor encompassing primary myelofibrosis (PMF) and post-polycythemia *vera* and post-essential thrombocythemia (ET) myelofibrosis (post-PV/ET MF) [1]. Most individuals with MPN-associated myelofibrosis have so-called *driver* mutations in *JAK2*, *CALR*, *or MPL.* Although the distribution of these mutations is similar in PMF and post-PV/ET MF, some data indicate that the impact of the type of *driver* mutation on prognosis differs [2]. Recently, non-driver mutations were reported to be associated with the initiation, progression, and prognosis of MPNs [3–6]. We analyzed whether the landscape of non-driver mutations differed among cohorts.

Forty-five subjects with PMF and 17 with post-PV/ET MF (13 post-PV MF and 4 post-ET MF) were studied. The subjects provided informed consent in compliance with the Declaration of Helsinki (The Ethics Committee of Institute of Hematology and Blood Diseases Hospital; No. KT2015010-EC-1). Diagnoses were based on the revised 2016 criteria of the World Health Organization (WHO) [7]. The median interval from diagnosis of PV or ET to post-PV/ET MF was 12 years (range, 5–30 years). Prognosis was evaluated with the Dynamic International Prognostic Scoring System (DIPSS) [8]. The cytogenetic findings were classified as favorable or unfavorable using the DIPSS-plus classification [9]. Subject-, disease-, and therapy-related variables are displayed in Additional file 1: Table S1.

Targeted gene sequencing was carried out upon diagnosis of myelofibrosis. Non-driver mutations were compared against expected patterns and categorized into oncogenic mutations, possibly oncogenic variants or unknown significance. Germline control DNA was used to ascertain the somatic mutations. To account for the absence of a matched control for some subjects, a *bespoke* variant selection pipeline was used*.* Details of the methods are described in the Additional file 2 and Additional file 1: Table S2–S3.

Forty-two subjects with PMF (93%) had ≥1 non-driver mutation. Two of the 17 subjects with no driver mutation in *JAK2* ^V617*F*^, *JAK2* exon 12, *CALR*, or *MPL* ^W515^ had mutations in *SH2B3* or *PIAS3* of the *JAK-STAT* signaling pathway, 13 had mutations in other genes, and 2 had no mutations. Non-driver mutations were detected in 16 subjects with post-PV/ET MF (94%). There was no difference in the median numbers of non-driver mutations in the subjects with PMFs vs. those with post-PV/ET MF (3; [range, 0–5] vs. 3; [range, 0–7]; *P* = 0.885). There was also no difference in the median numbers of non-driver mutations (3; [range, 0–5] vs. 3 [range, 0–5]; *P* = 0.668) in the subjects with PMF with and without *driver* mutations.

**Fig. 1:**
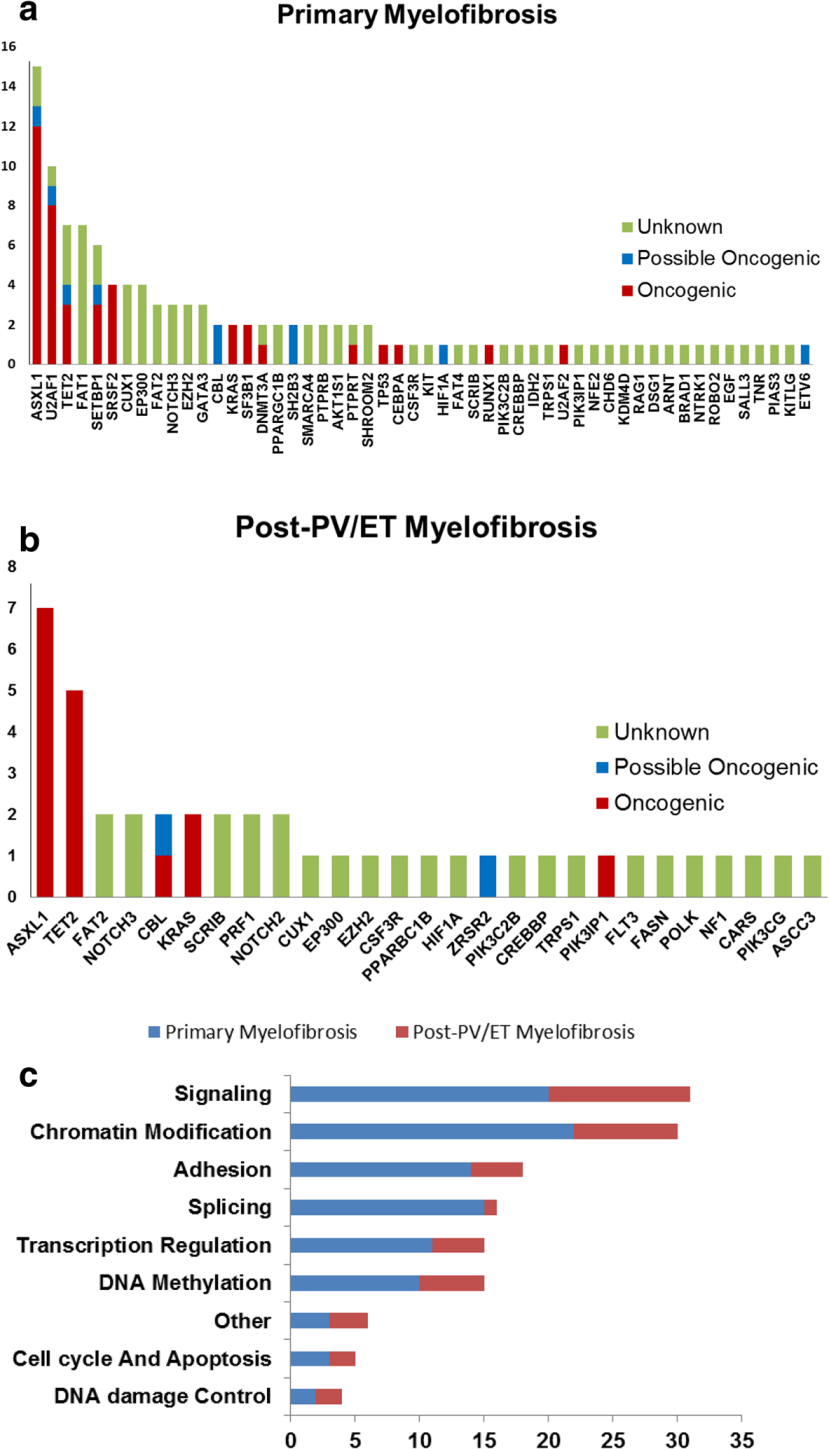
Mutational spectrum in primary myelofibrosis and post-PV/ET myelofibrosis. Frequency of non-driver mutations identified in the sequencing screen in 45 patients with PMF (**a**) and 17 patients with post-PV/ET MF (**b**). Frequency of gene mutations involved in common functional pathways (**c**)

The distribution of non-driver mutations in PMF vs. post-PV/ET MF is shown in Fig. 1a, b. Twelve mutations in non-driver genes were detected in >5% of the subjects with PMF, including *ASXL1* (*N* = 15), *U2AF1* (*N* = 10), *TET2* (*N* = 7), *FAT1* (*N* = 7), *SETBP1* (*N* = 6), *SRSF2* (*N* = 4), *CUX1* (*N* = 4), *EP300* (*N* = 4), *FAT2* (*N* = 3), *NOTCH3* (*N* = 3), *EZH2* (*N* = 3), and *GATA3* (*N* = 3). In subjects with post-PV/ET MF, *ASXL1* (*N* = 7) was the most frequent mutation, followed by *TET2* (*N* = 5).

Changes in non-driver genes were grouped into several functional pathways. The most frequent mutation targets were genes associated with signal transduction (*N* = 31), followed by chromatin modification (*N* = 30), adhesion (*N* = 18), RNA splicing (*N* = 16), transcription (*N* = 15), DNA methylation (*N* = 15), cell cycle/apoptosis (*N* = 5), and DNA damage control (*N* = 4; Fig. 1c). The mutation frequencies of the mRNA splicing genes were significantly different between the subjects with PMF and those with post-PV/ET (33 vs. 6%; *P* = 0.015). *U2AF1* mutations were significantly more common in the subjects with PMF than those with post-PV/ET MF (22 vs. 0; *P* = 0.008).

**Fig. 2:**
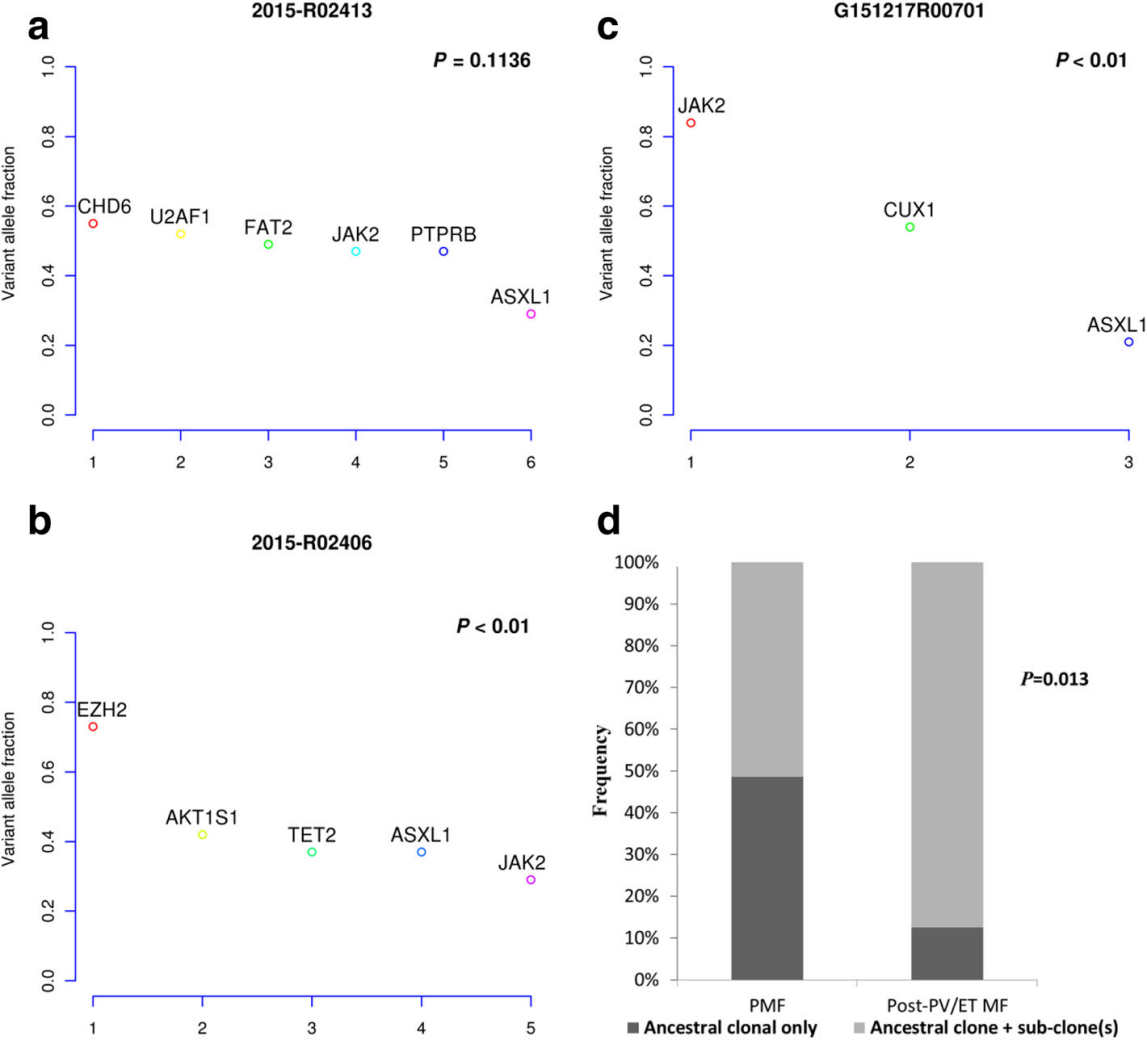
Clonal architecture in PMF and post-PV/ET MF. **a** In a representative patient with PMF, mutant *JAK2* co-occurs with non-driver mutations as ancestral mutations. **b** In a representative patient with PMF, mutant *JAK2* was a sub-clonal mutation and other non-driver mutations precede mutant *JAK2*. **c** In a representative patient with post-PV MF, mutant *JAK2* was the only ancestral mutation preceding other non-driver mutations. **d** More PMFs were classified as having an *ancestral clonal only* architecture than post-PV/ET MF

We used copy number-adjusted variant allelic frequencies (VAF) in each subject with a non-driver mutation to determine whether a mutation was in the ancestral clone or whether it arose in a sub-clone. Using statistically significant differences in VAFs, the subjects were classified as representing two different clonal architectures: (1) *ancestral clonal only*; or (2) *ancestral clone* and *sub-clone(s)*. Figure 2a–c shows data from three subjects. Frequencies of ancestral clonal only vs. ancestral clone and sub-clone(s) architecture were significantly different between the subjects with PMF and those with post-PV/ET MF (ancestral clonal only: 49 vs. 13%; *P* = 0.013; Fig. 2d). In the subjects with PMF, the *driver* and non-driver mutations were ancestral in 14 subjects (e.g., subject 2015-R02413 in Fig. 2a) and sub-clonal in 9 subjects (e.g., subject 2015-R02406 in Fig. 2b). Eleven subjects with post-PV/ET MFs had *JAK2* ^V617F^ as the only ancestral mutation (e.g., subject G121517R00701 in Fig. 2c).

In conclusion, we found differences in the non-driver mutation profile and the clonal architecture among the subjects with PMF compared with those with post-PV/ET MF. There was a significant difference in the frequency of the mRNA splicing genes among cohorts, similar to data reported by others [2, 10]. In individuals with PMF, approximately 30% of *driver* mutations co-occur with non-driver mutations as ancestral events, whereas approximately 20% of *driver* mutations were in sub-clones. In contrast, *driver* mutations occurred only in the ancestral clone in most subjects with post-PV/ET MF.

## Additional files

Additional file 1: Table S1. Clinical and laboratory features of 45 patients with PMF and 17 patients with post-PV/ET MF. **Table S2.** Gene list of the 190-gene NGS panel. **Table S3.** Two hundred and twenty-nine high-confidence variants sequenced by the 190-gene NGS panel. (DOCX 43 kb)

Additional file 2: Methods (DOCX 26 kb)

## Abbreviations

ET: Essential thrombocythemia
MPN: Myeloproliferative neoplasm
PMF: Primary myelofibrosis
PV: Polycythemia *vera*
VAF: Variant allelic frequency
