# Supplementary material for: Non-driver mutations in myeloproliferative neoplasm-associated myelofibrosis
Source: J Hematol Oncol. 2017 May 2;10:99. doi: 10.1186/s13045-017-0472-5 (PMC5414291; doi:10.1186/s13045-017-0472-5)
Supplement: Supplementary file 1 — Clinical and laboratory features of 45 patients with PMF and 17 patients with post-PV/ET MF. Table S2. Gene list of the 190-gene NGS panel. Table S3. Two hundred and twenty-nine high-confidence variants sequenced by the 190-gene NGS panel. (DOCX 43 kb) [file 13045_2017_472_MOESM1_ESM.docx]

**Table S1.** Clinical and laboratory features of 45 patients with PMF and 17 patients with post-PV/ET MF.

| **Variable** | **PMF** | **Post-PV/ET MF** | ***P*** |
| --- | --- | --- | --- |
| Age (y) median (range) | 55 (21-77) | 59 (46-83) | 0.051 |
| Age>65yrs; n (%) | 6 (13.3) | 6 (35.3) | 0.061 |
| Males n (%) | 31 (68.9) | 10 (58.8) | 0.455 |
| Hemoglobin (g/L) median (range) | 86 (36-158) | 118 (86-191) | 0.001 |
| HGB<100g/L; n(%) | 24 (53.3) | 4 (23.5) | 0.035 |
| WBC (x109/L) median (range) | 5 (0.5-52) | 12.3 (1.85-42) | 0.017 |
| WBC>25x109/L; n (%) | 4 (8.9) | 4 (23.5) | 0.144 |
| Platelets (x109/L) median (range) | 106 (4-960) | 257 (56-1044) | 0.004 |
| Platelets<100 x109/L; n (%) | 20 (44.4) | 3 (17.6) | 0.051 |
| Blood blast≥1%; n (%) | 14 (31.1) | 2 (11.8) | 0.101 |
| Constitutional symtoms; n (%) | 10 (22.2) | 4 (23.5) | 0.913 |
| Splenomagly from LCM (cm) median (range) | 3 (0-19.5) | 6 (0-20) | 0.161 |
| Splenomagly>5cm LCM; n (%) | 18 (40) | 9 (52.9) | 0.359 |
| Unfavorable Karyotype | 5 (11.9) | 0 | 0.091 |
| Fibrosis grade 2-3; n (%) | 40 (88.9) | 17 (100) | 0.066 |
| Driver mutations; n (%) |  |  | 0.202 |
| JAK2V617F | 22 (48.9) | 16 (94.1) | 0.001 |
| MPLW515 | 1 (2.2) | 0 |  |
| CALR | 5 (11.1) | 2 (11.7) |  |
| Triple-negative | 17 (37.8) | 0 |  |
| Number of non-driver mutation; n (%) |  |  | 0.929 |
| 0 | 3 (6.7) | 1 (5.9) |  |
| 1 | 9 (20) | 3 (17.6) |  |
| 2 | 9 (20) | 4 (23.5) |  |
| ≥3 | 24 (53.3) | 9 (53) |  |
| DIPSS risk group; n (%) |  |  |  |
| Low | 6 (13.3) |  |  |
| Int-1 | 25 (55.6) |  |  |
| Int-2 | 14 (31.1) |  |  |

**Table S2.** Gene list of the 190-gene NGS panel.

| AKT1 | C6 | EGLN1 | GATA3 | IRF8 | NFE2 | PIK3C3 | PTPRB | SMARCA4 | TRPC4 |
| --- | --- | --- | --- | --- | --- | --- | --- | --- | --- |
| AKT1S1 | CALR | EID1 | GCSF | JAK2 | NFKB2 | PIK3CA | PTPRT | SOCS1 | TRPS1 |
| AKT2 | CARD6 | EID2 | GDF15 | JUNB | NFKBIA | PIK3CB | RAG1 | SOCS2 | U2AF1 |
| AKT3 | CARS | EID3 | GSN | JUN-D | NOTCH2 | PIK3CD | RAG2 | SOCS3 | U2AF2 |
| AKTIP | CBL | EPO | HCN1 | KDM4D | NOTCH3 | PIK3CG | RASGRF1 | SOX2 | U2AF35 |
| AML1 | CCDC88A | EPOR | HEATR8 | KIAAO355 | NPM1 | PIK3IP1 | RBBP5 | SRSF2 | UTX |
| APC | CEBPA | ERG | HIF1A | KIF17 | NRAS | PIK3R1 | RCOR1 | STAG2 | VHL |
| ARID4A | CHD4 | ETV6 | HIF3A | KIT | NTRK1 | PIK3R2 | ROBO2 | STAT3 | VSP5 |
| ARNT | CHD6 | EVI1 | HINT1 | KRAS | NXF1 | PIK3R3 | RUNX1 | STAT5 | WT1 |
| ARNT2 | CREBBP | EZH2 | HOXA9 | L3MBTL | p300(EP300) | PIK3R4 | SALL3 | STK11 | ZFP36L1 |
| ARNTL | CSF3R | FASN | HSD17B4 | LNK(SH2B3) | PARP3 | PIK3R5 | SCF(KITLG) | SUZ12 | ZRSR2 |
| ARNTL2 | CSNK2A1 | FAT1 | HUWE1 | MAST4 | PAS1 | PIK3R6 | SCRIB | TET2 |  |
| ASSCC3 | CUX1 | FAT2 | IDH1 | MPL | PDE4C | POLK | SETBP1 | TGFB1 |  |
| ASXL1 | DLGAP2 | FAT4 | IDH2 | MYB | PIAS1 | PPARGC1A | SETD8 | TGFBR1 |  |
| BARD1 | DNMT3A | FGFRα | IDO1 | MYBL1 | PIAS2 | PPARGC1B | SF3A1 | TGFBR2 |  |
| BCL2 | DNMT3B | FLT3 | IFI30 | MYBL2 | PIAS3 | PPHLN1 | SF3B1 | THPO |  |
| BMI1 | DSG1 | FOXP1 | IKZF1 | MYC | PIAS4 | PRF1 | SGK2 | TNFA |  |
| BRAF | EED | FOXP2 | IL6 | MYCBP | PIK3AP1 | PRMT5 | SH2B3 | TNFRSF |  |
| BRCA2 | EGF | GATA1 | IL6R | MYPN | PIK3C2A | PTEN | SHROOM2 | TNR |  |
| BRD2 | EGFR | GATA2 | IRF4 | NF1 | PIK3C2B | PTPN11 | SMARCA2 | TP53 |  |

**Table S3.** Two hundred and twenty-nine high-confidence variants sequenced by the 190-gene NGS panel.

| **SAMPLE NUMBER** | **ID_VARIANT** | **Category** | **CHR** | **Type** | **Gene** | **EXON** | **c.** | **p.** | **Annotation** |
| --- | --- | --- | --- | --- | --- | --- | --- | --- | --- |
| 2015-R02417 | 353392 | PMF | 19 | Substitution | AKT1S1 | 2 | c.349G>A | p.G117R | Unknown |
| 2015-R02406 | 244814 | PMF | 19 | Substitution | AKT1S1 | 2 | c.349G>A | p.G117R | Unknown |
| 2015-R02736 | 391698 | PMF | 1 | Substitution | ARNT | 7 | c.659G>A | p.R220H | Unknown |
| C151217R00501 | 95415 | PET-MF | 6 | Substitution | ASCC3 | 9 | c.1595A>G | p.K532R | Unknown |
| C151217R01301 | 278617 | PPV-MF | 20 | Substitution | ASXL1 | 12 | c.2083C>T | p.Q695X | Oncogenic |
| G151217R01001 | 91690 | PPV-MF | 20 | Substitution | ASXL1 | 12 | c.2324T>A | p.L775X | Oncogenic |
| C151217R01001 | 53107 | PPV-MF | 20 | Insertion | ASXL1 | 12 | c.2024_2025insTGAG | p.P675fs | Oncogenic |
| G151217R00701 | 26519 | PPV-MF | 20 | Deletion | ASXL1 | 12 | c.2422delC | p.P808fs | Oncogenic |
| S3390 | 455630 | PMF | 20 | Substitution | ASXL1 | 12 | c.2324T>G | p.L775X | Oncogenic |
| S3390 | 455630 | PMF | 20 | Substitution | ASXL1 | 12 | c.3202C>T | p.R1068X | Oncogenic |
| S2889 | 449874 | PMF | 20 | Substitution | ASXL1 | 12 | c.1954G>A | p.G652S | Unknown |
| S2932 | 392214 | PMF | 20 | Substitution | ASXL1 | 12 | c.1954G>A | p.G652S | Unknown |
| 2015-R02409 | 386757 | PMF | 20 | Insertion | ASXL1 | 12 | c.2413insC | p.G804fs | Oncogenic |
| 2015-R02399 | 382626 | PMF | 20 | Insertion | ASXL1 | 12 | c.4122insG | p.V1374fs | Oncogenic |
| 2015-R02408 | 378712 | PMF | 20 | Substitution | ASXL1 | 7 | c.599G>T | p.G200V | Unknown |
| 2015-R02404 | 362003 | PMF | 20 | Insertion | ASXL1 | 11 | c.1618insC | p.K539fs | Oncogenic |
| 2015-R02416 | 357292 | PMF | 20 | Insertion | ASXL1 | 12 | c.1927insG | p.G642fs | Oncogenic |
| G151217R01301 | 323101 | PMF | 20 | Deletion | ASXL1 | 12 | c.1888_1910del | p.H630fs | Oncogenic |
| 2015-R02403 | 320860 | PMF | 20 | Insertion | ASXL1 | 12 | c.1772insA | p.Y591_Q592delinsX | Oncogenic |
| 2015-R02390 | 300877 | PMF | 20 | Insertion | ASXL1 | 12 | c.1927insG | p.G642fs | Oncogenic |
| 2015-R02413 | 260731 | PMF | 20 | Deletion | ASXL1 | 12 | c.1888_1910del | p.H630fs | Oncogenic |
| 2015-R02396 | 248948 | PMF | 20 | Insertion | ASXL1 | 12 | c.1927insG | p.G642fs | Oncogenic |
| 2015-R02406 | 244814 | PMF | 20 | Substitution | ASXL1 | 12 | c.1954G>A | p.G652S | Unknown |
| 2015-R02406 | 244814 | PMF | 20 | Deletion | ASXL1 | 12 | c.2422delC | p.P808fs | Possible oncogenic |
| 2015-R02414 | 172079 | PMF | 20 | Insertion | ASXL1 | 12 | c.1927insG | p.G642fs | Oncogenic |
| C151217R00101 | 142803 | PET-MF | 20 | Substitution | ASXL1 | 12 | c.2122C>T | p.Q708X | Oncogenic |
| C151217R00501 | 95415 | PET-MF | 20 | Deletion | ASXL1 | 12 | c.2323delT | p.L775X | Oncogenic |
| C151217R00501 | 95415 | PET-MF | 20 | Deletion | ASXL1 | 12 | c.2463_2467delTACAT | p.D821fs | Oncogenic |
| C151217R00201 | 62445 | PET-MF | 20 | Deletion | ASXL1 | 12 | c.2384delC | p.S795fs | Oncogenic |
| 2015-R02736 | 391698 | PMF | 2 | Substitution | BARD1 | 2 | c.176G>A | p.C59Y | Unknown |
| C151217R01501 | 87837 | PPV-MF | 19 | Substitution | CALR | 9 | c.1142A>C | p.E381A | Possible oncogenic |
| 2015-R02424 | 351722 | PMF | 19 | Deletion | CALR | 9 | c.1123delA | p.K375fs | Oncogenic |
| C151217R00201 | 62445 | PET-MF | 19 | Insertion | CALR | 9 | c.1154_1155insTTGTC | p.K385fs | Oncogenic |
| 2015-R02422 | 379887 | PMF | 19 | Deletion | CALR | 9 | [c.1092_1143del52](http://cancer.sanger.ac.uk/cosmic/mutation/overview?id=1738055) | p.L367fs*46 | Oncogenic |
| 2015-R02396 | 248948 | PMF | 19 | Deletion | CALR | 9 | c.1092_1143del52 | p.L367fs*46 | Oncogenic |
| 2015-R02409 | 386757 | PMF | 19 | Insertion | CALR | 9 | c.1154_1155insTTGTC | p.K385fs*47 | Oncogenic |
| 2015-R02401 | 254111 | PMF | 19 | Deletion | CALR | 9 | c.1092_1143del52 | p.L367fs*46 | Oncogenic |
| G151217R00501 | 147454 | PPV-MF | 11 | Substitution | CARS | 9 | c.795T>G | p.N265K | Unknown |
| C151217R01301 | 278617 | PPV-MF | 11 | Substitution | CBL | 9 | c.1259G>A | p.R420Q | Oncogenic |
| 2015-R02423 | 367645 | PMF | 11 | Substitution | CBL | 11 | c.1858C>T | p.L620F | Unknown |
| 2015-R02396 | 248948 | PMF | 11 | Substitution | CBL | 8 | c.1111T>A | p.Y371N | Possible oncogenic |
| C151217R00201 | 62445 | PET-MF | 11 | Substitution | CBL | 8 | c.1111T>G | p.Y371D | Possible oncogenic |
| S3479 | 455551 | PMF | 19 | Insertion | CEBPA | 1 | c.68insC | p.P23fs | Oncogenic |
| 2015-R02413 | 260731 | PMF | 20 | Substitution | CHD6 | 32 | c.6215G>A | p.R2072Q | Unknown |
| 2015-R02394 | 349267 | PMF | 16 | Substitution | CREBBP | 2 | c.760G>A | p.A254T | Unknown |
| C151217R00101 | 142803 | PET-MF | 16 | Substitution | CREBBP | 31 | c.6983C>T | p.S2328L | Unknown |
| G151217R01001 | 91690 | PPV-MF | 1 | Substitution | CSF3R | 17 | c.2197C>A | p.P733T | Unknown |
| S2932 | 392214 | PMF | 1 | Substitution | CSF3R | 17 | c.2197C>A | p.P733T | Unknown |
| G151217R00701 | 26519 | PPV-MF | 7 | Substitution | CUX1 | 18 | c.1678G>A | p.E560K | Unknown |
| 2015-R02423 | 367645 | PMF | 7 | Substitution | CUX1 | 20 | c.3161C>T | p.S1054L | Unknown |
| 2015-R02394 | 349267 | PMF | 7 | Substitution | CUX1 | 20 | c.3161C>T | p.S1054L | Unknown |
| 2015-R02412 | 338420 | PMF | 7 | Substitution | CUX1 | 18 | c.2332C>T | p.P778S | Unknown |
| 2015-R02392 | 285326 | PMF | 7 | Substitution | CUX1 | 17 | c.1588A>C | p.K530Q | Unknown |
| S3479 | 455551 | PMF | 2 | Substitution | DNMT3A | 22 | c.2578T>C | p.W860R | Oncogenic |
| S2932 | 392214 | PMF | 2 | Substitution | DNMT3A | 14 | c.1640T>A | p.L547H | Unknown |
| 2015-R02736 | 391698 | PMF | 18 | Substitution | DSG1 | 5 | c.2134C>T | p.R712C | Unknown |
| 2015-R02424 | 351722 | PMF | 4 | Substitution | EGF | 1 | c.46A>C | p.S16R | Unknown |
| C151217R00801 | 301882 | PPV-MF | 22 | Substitution | EP300 | 6 | c.1519A>G | p.S507G | Unknown |
| 2015-R02399 | 382626 | PMF | 22 | Substitution | EP300 | 6 | c.1519A>G | p.S507G | Unknown |
| 2015-R02425 | 370665 | PMF | 22 | Substitution | EP300 | 6 | c.1519A>G | p.S507G | Unknown |
| G151217R01301 | 323101 | PMF | 22 | Substitution | EP300 | 6 | c.1519A>G | p.S507G | Unknown |
| 2015-R02396 | 248948 | PMF | 22 | Substitution | EP300 | 31 | c.5957C>T | p.P1986L | Unknown |
| 2015-R02403 | 320860 | PMF | 12 | Substitution | ETV6 | 6 | c.1057C>T | p.R353W | Unknown |
| C151217R01001 | 53107 | PPV-MF | 7 | Substitution | EZH2 | 16 | c.1854G>T | p.L618F | Unknown |
| 2015-R02416 | 357292 | PMF | 7 | Substitution | EZH2 | 4 | c.257A>T | p.E86V | Unknown |
| 2015-R02406 | 244814 | PMF | 7 | Substitution | EZH2 | 11 | c.1373G>A | p.R458Q | Unknown |
| 2015-R02406 | 244814 | PMF | 7 | Substitution | EZH2 | 12 | c.1505G>A | p.R502Q | Unknown |
| 2015-R02414 | 172079 | PMF | 7 | Substitution | EZH2 | 4 | c.275T>G | p.I92S | Unknown |
| G151217R01001 | 91690 | PPV-MF | 17 | Substitution | FASN | 29 | c.5050G>A | p.A1684T | Unknown |
| S2834 | 448810 | PMF | 4 | Substitution | FAT1 | 10 | c.7130C>T | p.T2377M | Unknown |
| 2015-R02735 | 400740 | PMF | 4 | Substitution | FAT1 | 27 | c.13652C>G | p.A4551G | Unknown |
| S2932 | 392214 | PMF | 4 | Substitution | FAT1 | 10 | c.8465T>C | p.L2822P | Unknown |
| S2879 | 385178 | PMF | 4 | Substitution | FAT1 | 25 | c.12653A>G | p.D4218G | Unknown |
| 2015-R02399 | 382626 | PMF | 4 | Substitution | FAT1 | 2 | c.3178G>C | p.D1060H | Unknown |
| 2015-R02425 | 370665 | PMF | 4 | Substitution | FAT1 | 10 | c.8465T>C | p.L2822P | Unknown |
| 2015-R02392 | 285326 | PMF | 4 | Substitution | FAT1 | 27 | c.13652C>G | p.A4551G | Unknown |
| C151217R00801 | 301882 | PPV-MF | 5 | Substitution | FAT2 | 9 | c.6476T>C | p.V2159A | Unknown |
| C151217R01301 | 278617 | PPV-MF | 5 | Substitution | FAT2 | 12 | c.9224G>A | p.R3075Q | Unknown |
| 2015-R02390 | 300877 | PMF | 5 | Substitution | FAT2 | 1 | c.1310C>T | p.P437L | Unknown |
| 2015-R02413 | 260731 | PMF | 5 | Substitution | FAT2 | 9 | c.6476T>C | p.V2159A | Unknown |
| 2015-R02414 | 172079 | PMF | 5 | Substitution | FAT2 | 20 | c.11729A>T | p.E3910V | Unknown |
| 2015-R02420 | 367855 | PMF | 4 | Substitution | FAT4 | 9 | c.11693C>T | p.A3898V | Unknown |
| C151217R00501 | 95415 | PET-MF | 13 | Substitution | FLT3 | 9 | c.1073A>T | p.D358V | Unknown |
| S2889 | 449874 | PMF | 10 | Insertion | GATA3 | 6 | c.1126insA | p.C375fs | Unknown |
| S2879 | 385178 | PMF | 10 | Insertion | GATA3 | 6 | c.1126insA | p.C375fs | Unknown |
| 2015-R02402 | 330340 | PMF | 10 | Substitution | GATA3 | 3 | c.604C>T | p.R202C | Unknown |
| 2015-R02422 | 379887 | PMF | 14 | Substitution | HIF1A | 12 | c.1762G>A | p.A588T | Possible oncogenic |
| C151217R00101 | 142803 | PET-MF | 14 | Substitution | HIF1A | 12 | c.1834G>A | p.A612T | Unknown |
| C150910R00201 | 338942 | PMF | 15 | Substitution | IDH2 | 2 | c.148G>A | p.V50M | Unknown |
| C151217R01201 | 353979 | PPV-MF | 9 | Substitution | JAK2 | 14 | c.1849G>T | p.V617F | Oncogenic |
| C151217R00601 | 336444 | PPV-MF | 9 | Deletion | JAK2 | 12 | c.1622_1627delGAAATG | p.541_543del | Oncogenic |
| C151217R00801 | 301882 | PPV-MF | 9 | Substitution | JAK2 | 14 | c.1849G>T | p.V617F | Oncogenic |
| C151217R01401 | 296837 | PPV-MF | 9 | Deletion | JAK2 | 12 | c.1612_1613delCA | p.H538fs | Oncogenic |
| C151217R01401 | 296837 | PPV-MF | 9 | Insertion | JAK2 | 12 | c.1616_1617insTT | p.K539fs | Oncogenic |
| C151217R01401 | 296837 | PPV-MF | 9 | Substitution | JAK2 | 12 | c.1614C>A | p.H538Q | Oncogenic |
| C151217R01401 | 296837 | PPV-MF | 9 | Substitution | JAK2 | 12 | c.1615A>T | p.K539X | Oncogenic |
| C151217R01301 | 278617 | PPV-MF | 9 | Substitution | JAK2 | 14 | c.1849G>T | p.V617F | Oncogenic |
| C151217R01101 | 250642 | PPV-MF | 9 | Substitution | JAK2 | 14 | c.1849G>T | p.V617F | Oncogenic |
| G151217R00801 | 219652 | PPV-MF | 9 | Substitution | JAK2 | 14 | c.1849G>T | p.V617F | Oncogenic |
| C151217R00901 | 198419 | PPV-MF | 9 | Substitution | JAK2 | 14 | c.1849G>T | p.V617F | Oncogenic |
| G151217R00501 | 147454 | PPV-MF | 9 | Substitution | JAK2 | 14 | c.1849G>T | p.V617F | Oncogenic |
| G151217R01001 | 91690 | PPV-MF | 9 | Substitution | JAK2 | 14 | c.1849G>T | p.V617F | Oncogenic |
| C151217R01501 | 87837 | PPV-MF | 9 | Substitution | JAK2 | 14 | c.1849G>T | p.V617F | Oncogenic |
| C151217R01001 | 53107 | PPV-MF | 9 | Substitution | JAK2 | 14 | c.1849G>T | p.V617F | Oncogenic |
| G151217R00701 | 26519 | PPV-MF | 9 | Substitution | JAK2 | 14 | c.1849G>T | p.V617F | Oncogenic |
| S3390 | 455630 | PMF | 9 | Substitution | JAK2 | 14 | c.1849G>T | p.V617F | Oncogenic |
| S2973 | 450824 | PMF | 9 | Substitution | JAK2 | 14 | c.1849G>T | p.V617F | Oncogenic |
| S2889 | 449874 | PMF | 9 | Substitution | JAK2 | 14 | c.1849G>T | p.V617F | Oncogenic |
| S2834 | 448810 | PMF | 9 | Substitution | JAK2 | 14 | c.1849G>T | p.V617F | Oncogenic |
| S2932 | 392214 | PMF | 9 | Substitution | JAK2 | 14 | c.1849G>T | p.V617F | Oncogenic |
| 2015-R02736 | 391698 | PMF | 9 | Substitution | JAK2 | 14 | c.1849G>T | p.V617F | Oncogenic |
| 2015-R02408 | 378712 | PMF | 9 | Substitution | JAK2 | 14 | c.1849G>T | p.V617F | Oncogenic |
| 2015-R02421 | 374060 | PMF | 9 | Substitution | JAK2 | 14 | c.1849G>T | p.V617F | Oncogenic |
| 2015-R02423 | 367645 | PMF | 9 | Substitution | JAK2 | 14 | c.1849G>T | p.V617F | Oncogenic |
| C150910R00301 | 358488 | PMF | 9 | Substitution | JAK2 | 14 | c.1849G>T | p.V617F | Oncogenic |
| C150910R00401 | 354766 | PMF | 9 | Substitution | JAK2 | 9 | c.1174G>A | p.V392M | Unknown |
| 2015-R02415 | 339831 | PMF | 9 | Substitution | JAK2 | 14 | c.1849G>T | p.V617F | Oncogenic |
| C150910R00201 | 338942 | PMF | 9 | Substitution | JAK2 | 14 | c.1849G>T | p.V617F | Oncogenic |
| 2015-R02402 | 330340 | PMF | 9 | Substitution | JAK2 | 14 | c.1849G>T | p.V617F | Oncogenic |
| G151217R01301 | 323101 | PMF | 9 | Substitution | JAK2 | 14 | c.1849G>T | p.V617F | Oncogenic |
| 2015-R02403 | 320860 | PMF | 9 | Substitution | JAK2 | 14 | c.1849G>T | p.V617F | Oncogenic |
| 2015-R02400 | 305415 | PMF | 9 | Substitution | JAK2 | 14 | c.1849G>T | p.V617F | Oncogenic |
| 2015-R02398 | 293599 | PMF | 9 | Substitution | JAK2 | 14 | c.1849G>T | p.V617F | Oncogenic |
| 2015-R02392 | 285326 | PMF | 9 | Substitution | JAK2 | 14 | c.1849G>T | p.V617F | Oncogenic |
| 2015-R02413 | 260731 | PMF | 9 | Substitution | JAK2 | 14 | c.1849G>T | p.V617F | Oncogenic |
| 2015-R02406 | 244814 | PMF | 9 | Substitution | JAK2 | 14 | c.1849G>T | p.V617F | Oncogenic |
| C150910R00101 | 243517 | PMF | 9 | Substitution | JAK2 | 14 | c.1849G>T | p.V617F | Oncogenic |
| 2015-R02407 | 158502 | PMF | 9 | Substitution | JAK2 | 14 | c.1849G>T | p.V617F | Oncogenic |
| C151217R00401 | 256971 | PET-MF | 9 | Substitution | JAK2 | 14 | c.1849G>T | p.V617F | Oncogenic |
| C151217R00101 | 142803 | PET-MF | 9 | Substitution | JAK2 | 14 | c.1849G>T | p.V617F | Oncogenic |
| C151217R00501 | 95415 | PET-MF | 9 | Substitution | JAK2 | 14 | c.1849G>T | p.V617F | Oncogenic |
| C150910R00201 | 338942 | PMF | 11 | Substitution | KDM4D | 3 | c.175G>C | p.A59P | Unknown |
| 2015-R02736 | 391698 | PMF | 4 | Substitution | KIT | 16 | c.2263G>A | p.A755T | Unknown |
| 2015-R02422 | 379887 | PMF | 12 | Substitution | KITLG | 3 | c.160A>G | p.T54A | Unknown |
| C151217R00601 | 336444 | PPV-MF | 12 | Substitution | KRAS | 2 | c.34G>A | p.G12S | Oncogenic |
| 2015-R02399 | 382626 | PMF | 12 | Substitution | KRAS | 2 | c.53C>A | p.A18D | Oncogenic |
| 2015-R02416 | 357292 | PMF | 12 | Substitution | KRAS | 2 | c.34G>C | p.G12R | Oncogenic |
| C151217R00101 | 142803 | PET-MF | 12 | Substitution | KRAS | 2 | c.34G>A | p.G12S | Oncogenic |
| C151217R01401 | 296837 | PPV-MF | 1 | Substitution | MPL | 7 | c.1120A>G | p.T374A | Unknown |
| G151217R00801 | 219652 | PPV-MF | 1 | Substitution | MPL | 7 | c.1120A>G | p.T374A | Unknown |
| 2015-R02415 | 339831 | PMF | 1 | Substitution | MPL | 6 | c.962G>A | p.R321Q | Unknown |
| 2015-R02414 | 172079 | PMF | 1 | Substitution | MPL | 10 | c.1543T>G | p.W515G | Oncogenic |
| G151217R00501 | 147454 | PPV-MF | 17 | Substitution | NF1 | 17 | c.1933A>G | p.M645V | Unknown |
| 2015-R02414 | 172079 | PMF | 12 | Substitution | NFE2 | 3 | c.934C>T | p.R312C | Unknown |
| G151217R01001 | 91690 | PPV-MF | 1 | Substitution | NOTCH2 | 1 | c.8C>T | p.A3V | Unknown |
| G151217R01001 | 91690 | PPV-MF | 1 | Substitution | NOTCH2 | 1 | c.7G>T | p.A3S | Unknown |
| C151217R00101 | 142803 | PET-MF | 1 | Substitution | NOTCH2 | 28 | c.5065A>T | p.I1689F | Unknown |
| C151217R01401 | 296837 | PPV-MF | 19 | Substitution | NOTCH3 | 22 | c.3523C>T | p.R1175W | Unknown |
| 2015-R02424 | 351722 | PMF | 19 | Substitution | NOTCH3 | 24 | c.4348G>A | p.A1450T | Unknown |
| 2015-R02420 | 367855 | PMF | 19 | Substitution | NOTCH3 | 22 | c.3523C>T | p.R1175W | Unknown |
| 2015-R02401 | 254111 | PMF | 19 | Insertion | NOTCH3 | 33 | c.6102insC | p.G2035fs | Unknown |
| C151217R00101 | 142803 | PET-MF | 19 | Substitution | NOTCH3 | 22 | c.3523C>T | p.R1175W | Unknown |
| 2015-R02423 | 367645 | PMF | 1 | Substitution | NTRK1 | 3 | c.295G>A | p.V99M | Unknown |
| 2015-R02735 | 400740 | PMF | 1 | Substitution | PIAS3 | 14 | c.1783A>C | p.S595R | Unknown |
| C151217R01301 | 278617 | PPV-MF | 1 | Substitution | PIK3C2B | 19 | c.2812C>T | p.R938C | Unknown |
| 2015-R02394 | 349267 | PMF | 1 | Substitution | PIK3C2B | 20 | c.3023G>A | p.R1008Q | Unknown |
| C151217R00401 | 256971 | PET-MF | 7 | Substitution | PIK3CG | 11 | c.3062G>A | p.R1021H | Unknown |
| C151217R01001 | 53107 | PPV-MF | 22 | Substitution | PIK3IP1 | 3 | c.193G>A | p.G65S | Unknown |
| C150910R00101 | 243517 | PMF | 22 | Substitution | PIK3IP1 | 3 | c.193G>A | p.G65S | Unknown |
| C151217R00901 | 198419 | PPV-MF | 5 | Substitution | POLK | 4 | c.382A>G | p.I128V | Unknown |
| 2015-R02422 | 379887 | PMF | 5 | Substitution | PPARGC1B | 4 | c.709G>A | p.G237S | Unknown |
| 2015-R02425 | 370665 | PMF | 5 | Substitution | PPARGC1B | 8 | c.2551G>A | p.A851T | Unknown |
| C151217R00201 | 62445 | PET-MF | 5 | Substitution | PPARGC1B | 10 | c.2734G>T | p.D912Y | Unknown |
| G151217R00801 | 219652 | PPV-MF | 10 | Substitution | PRF1 | 3 | c.1153C>T | p.R385W | Unknown |
| C151217R00901 | 198419 | PPV-MF | 10 | Substitution | PRF1 | 3 | c.1228C>T | p.R410W | Unknown |
| 2015-R02417 | 353392 | PMF | 12 | Substitution | PTPRB | 24 | c.4904C>T | p.P1635L | Unknown |
| 2015-R02413 | 260731 | PMF | 12 | Substitution | PTPRB | 24 | c.4904C>T | p.P1635L | Unknown |
| 2015-R02419 | 350398 | PMF | 20 | Substitution | PTPRT | 22 | c.3019G>T | p.V1007F | Possible oncogenic |
| 2015-R02392 | 285326 | PMF | 20 | Substitution | PTPRT | 7 | c.982C>T | p.R328C | Unknown |
| 2015-R02418 | 352530 | PMF | 11 | Substitution | RAG1 | 2 | c.1573C>T | p.P525S | Unknown |
| 2015-R02420 | 367855 | PMF | 3 | Substitution | ROBO2 | 12 | c.1739A>G | p.Y580C | Unknown |
| 2015-R02418 | 352530 | PMF | 21 | Substitution | RUNX1 | 4 | c.703C>T | p.Q235X | Oncogenic |
| 2015-R02424 | 351722 | PMF | 18 | Substitution | SALL3 | 2 | c.3265G>A | p.G1089R | Unknown |
| C151217R01001 | 53107 | PPV-MF | 8 | Substitution | SCRIB | 10 | c.1036C>T | p.R346C | Unknown |
| 2015-R02416 | 357292 | PMF | 8 | Deletion | SCRIB | 2 | c.167delT | p.F56fs | Unknown |
| C151217R00501 | 95415 | PET-MF | 8 | Substitution | SCRIB | 23 | c.3367G>A | p.A1123T | Unknown |
| 2015-R02422 | 379887 | PMF | 18 | Substitution | SETBP1 | 4 | c.2612T>C | p.I871T | Oncogenic |
| 2015-R02423 | 367645 | PMF | 18 | Substitution | SETBP1 | 6 | c.4398G>T | p.E1466D | Unknown |
| 2015-R02403 | 320860 | PMF | 18 | Substitution | SETBP1 | 4 | c.1387C>T | p.R463C | Unknown |
| 2015-R02390 | 300877 | PMF | 18 | Deletion | SETBP1 | 3 | c.494_508del | p.165_170del | Unknown |
| 2015-R02392 | 285326 | PMF | 18 | Substitution | SETBP1 | 4 | c.2602G>A | p.D868N | Oncogenic |
| 2015-R02414 | 172079 | PMF | 18 | Substitution | SETBP1 | 4 | c.2602G>A | p.D868N | Oncogenic |
| S3390 | 455630 | PMF | 2 | Substitution | SF3B1 | 15 | c.2098A>G | p.K700E | Oncogenic |
| 2015-R02392 | 285326 | PMF | 2 | Substitution | SF3B1 | 14 | c.1998G>T | p.K666N | Oncogenic |
| 2015-R02420 | 367855 | PMF | 12 | Substitution | SH2B3 | 2 | c.724C>T | p.P242S | Possible oncogenic |
| C150910R00101 | 243517 | PMF | 12 | Substitution | SH2B3 | 2 | c.724C>T | p.P242S | Possible oncogenic |
| C150910R00101 | 243517 | PMF | 12 | Substitution | SH2B3 | 7 | c.1000G>A | p.A334T | Possible oncogenic |
| 2015-R02419 | 350398 | PMF | X | Substitution | SHROOM2 | 4 | c.1549C>T | p.R517C | Unknown |
| 2015-R02398 | 293599 | PMF | X | Substitution | SHROOM2 | 4 | c.2693C>T | p.A898V | Unknown |
| C151217R00701 | 293599 | PMF | X | Substitution | SHROOM2 | 10 | c.4636A>C | p.K1546Q | Unknown |
| 2015-R02421 | 374060 | PMF | 19 | Substitution | SMARCA4 | 32 | c.4711C>T | p.R1571W | Unknown |
| 2015-R02416 | 357292 | PMF | 19 | Substitution | SMARCA4 | 5 | c.878C>T | p.A293V | Unknown |
| 2015-R02399 | 382626 | PMF | 17 | Substitution | SRSF2 | 1 | c.284C>G | p.P95R | Oncogenic |
| G151217R01301 | 323101 | PMF | 17 | Substitution | SRSF2 | 1 | c.284C>A | p.P95H | Oncogenic |
| 2015-R02403 | 320860 | PMF | 17 | Substitution | SRSF2 | 1 | c.284C>A | p.P95H | Oncogenic |
| 2015-R02396 | 248948 | PMF | 17 | Substitution | SRSF2 | 1 | c.284C>A | p.P95H | Oncogenic |
| C151217R00801 | 301882 | PPV-MF | 4 | Deletion | TET2 | 3 | c.3077_3081delAGACC | p.E1026fs | Oncogenic |
| C151217R01301 | 278617 | PPV-MF | 4 | Insertion | TET2 | 3 | c.2490insA | p.I830fs | Oncogenic |
| C151217R01301 | 278617 | PPV-MF | 4 | Insertion | TET2 | 3 | c.2996insA: | p.E999fs | Oncogenic |
| C151217R00901 | 198419 | PPV-MF | 4 | Substitution | TET2 | 3 | c.1681A>T | p.K561X | Oncogenic |
| C151217R01501 | 87837 | PPV-MF | 4 | Substitution | TET2 | 3 | c.2440C>T | p.R814C | Oncogenic |
| S3390 | 455630 | PMF | 4 | Substitution | TET2 | 3 | c.972A>C | p.Q324H | Unknown |
| S2973 | 450824 | PMF | 4 | Substitution | TET2 | 3 | c.574T>C | p.Y192H | Unknown |
| S2973 | 450824 | PMF | 4 | Substitution | TET2 | 3 | c.972A>C | p.Q324H | Unknown |
| 2015-R02409 | 386757 | PMF | 4 | Insertion | TET2 | 3 | c.1821insA | p.G607fs | Possible oncogenic |
| 2015-R02421 | 374060 | PMF | 4 | Substitution | TET2 | 3 | c.3116C>T | p.S1039L | Unknown |
| C150910R00201 | 338942 | PMF | 4 | Substitution | TET2 | 3 | c.2604T>G | p.F868L | Oncogenic |
| 2015-R02400 | 305415 | PMF | 4 | Substitution | TET2 | 7 | c.3893G>A | p.C1298Y | Oncogenic |
| 2015-R02406 | 244814 | PMF | 4 | Substitution | TET2 | 3 | c.2839C>T | p.Q947X | Oncogenic |
| 2015-R02406 | 244814 | PMF | 4 | Substitution | TET2 | 10 | c.4457C>G | p.S1486X | Oncogenic |
| 2015-R02406 | 244814 | PMF | 4 | Substitution | TET2 | 3 | c.2839C>T | p.Q947X | Oncogenic |
| 2015-R02406 | 244814 | PMF | 4 | Substitution | TET2 | 10 | c.4457C>G | p.S1486X | Oncogenic |
| C151217R00101 | 142803 | PET-MF | 4 | Substitution | TET2 | 3 | c.2604T>G | p.F868L | Oncogenic |
| 2015-R02415 | 339831 | PMF | 1 | Deletion | TNR | 4 | c.637delG | p.V213fs | Unknown |
| S3479 | 455551 | PMF | 17 | Substitution | TP53 | 8 | c.818G>A | p.R273H | Oncogenic |
| G151217R00501 | 147454 | PPV-MF | 8 | Substitution | TRPS1 | 2 | c.515A>G | p.Q172R | Unknown |
| 2015-R02412 | 338420 | PMF | 8 | Substitution | TRPS1 | 2 | c.515A>G | p.Q172R | Unknown |
| S2932 | 392214 | PMF | 21 | Substitution | U2AF1 | 2 | c.101C>T | p.S34F | Oncogenic |
| S2879 | 385178 | PMF | 21 | Substitution | U2AF1 | 2 | c.101C>T | p.S34F | Oncogenic |
| 2015-R02422 | 379887 | PMF | 21 | Substitution | U2AF1 | 2 | c.101C>T | p.S34F | Oncogenic |
| 2015-R02404 | 362003 | PMF | 21 | Deletion | U2AF1 | 6 | c.569_583delAGATTAACCAGGACA | p.190_195del | Unknown |
| 2015-R02417 | 353392 | PMF | 21 | Deletion | U2AF1 | 4 | c.248_250delAGA | p.83_84del | Possible oncogenic |
| 2015-R02418 | 352530 | PMF | 21 | Substitution | U2AF1 | 2 | c.101C>A | p.S34Y | Oncogenic |
| C150910R00201 | 338942 | PMF | 21 | Substitution | U2AF1 | 2 | c.101C>T | p.S34F | Oncogenic |
| 2015-R02402 | 330340 | PMF | 21 | Substitution | U2AF1 | 2 | c.101C>A | p.S34Y | Oncogenic |
| 2015-R02390 | 300877 | PMF | 21 | Substitution | U2AF1 | 6 | c.470A>C | p.Q157P | Oncogenic |
| 2015-R02413 | 260731 | PMF | 21 | Substitution | U2AF1 | 2 | c.101C>A | p.S34Y | Oncogenic |
| 2015-R02402 | 330340 | PMF | 19 | Substitution | U2AF2 | 6 | c.559T>G | p.L187V | Oncogenic |
| C151217R01201 | 353979 | PPV-MF | X | Substitution | ZRSR2 | 10 | c.883C>T | p.R295X | Possible oncogenic |
| 2015-R02411 | 339966 | PMF | No mutation | | | | | | |
| 2015-R02393 | 204076 | PMF | No mutation | | | | | | |
