# Supplementary material for: Non-driver mutations in myeloproliferative neoplasm-associated myelofibrosis
Source: J Hematol Oncol. 2017 May 2;10:99. doi: 10.1186/s13045-017-0472-5 (PMC5414291; doi:10.1186/s13045-017-0472-5)
Supplement: Supplementary file 2 — Methods (DOCX 26 kb) [file 13045_2017_472_MOESM2_ESM.docx]

**Supplementary Material and Methods**

**Targeted gene sequencing**

DNAs from bone marrow aspirates were extracted and prepared for sequencing. Each DNA sample is quantiﬁed by agarose gel electrophoresis and Nanodrop (Thermo). Libraries were prepared using Illumina standard protocol. The amplified DNA was captured with a 190 MPN related Gene Panel using biotinylated oligo-probes (MyGenostics GenCap Enrichment technologies). The capture experiment was conducted according to manufacturer’s protocol. Illumina utilizes a unique "bridged" amplification reaction that occurs on the surface of the flow cell. A flow cell containing millions of unique clusters is loaded into the HiSeq 2000 for automated cycles of extension and imaging. Illumina's Sequencing-by-Synthesis utilizes four proprietary nucleotides possessing reversible fluorophore and termination properties. Each sequencing cycle occurs in the presence of all four nucleotides leading to higher accuracy than methods where only one nucleotide is present in the reaction mix at a time. This cycle is repeated, one base at a time, generating a series of images each representing a single base extension at a specific cluster.

Sanger sequencing for *CALR* exon 9 mutations was performed as previously reported [1].

**Basic bioinformatics analysis**

For nuclear gene sequencing analysis, high-quality reads were retrieved from raw reads by filtering out the low quality reads and adaptor sequences using the Solexa QA package and the cutadapt program (<http://code.google.com/p/cutadapt/>), respectively. SOAPaligner program was then used to align the clean read sequences to the human reference genome (hg19).

After the PCR duplicates were removed by the Picard software, the SNPs was firstly identified using the SOAPsnp program (<http://soap.genomics.org.cn/soapsnp.html>). Subsequently, we realigned the reads to the reference genome using BWA and identified the insertions or deletions (InDels) using the GATK program (<http://www.broadinstitute.org/gsa/wiki/index.php/Home_Page>). The identified SNPs and InDels were annotated using the Exome-assistant program (<http://122.228.158.106/exomeassistant>). MagicViewer was used to view the short read alignment and validate the candidate SNPs and InDels.

**Sequence variant annotation**

Once low confidence (Depth <5 or VAF <2%) or likely polymorphisms were removed from the dataset, each high confidence variant was annotated by 1000 Genomes, ESP6500, Inhouse, PolyPhen, SIFT and COSMIC to determine pathogenicity. To account for the absence of matched control, a bespoke variant selection pipeline from **Ref.2** was applied. The pipeline is detailed in the SUPPLEMENTARY MATERIAL AND METHODS **of Ref.2.**

Each variant was annotated as oncogenic, possible oncogenic or unknown in accordance to prior evidence in the literature in respect to the variants or genes association with myeloid disease.

**a. Oncogenic**

• Known myeloid malignancies related oncogenic variants previously reported in the literature;

• Truncating variants (nonsense mutations, essential splice mutations or frameshift indels) in genes implicated in myeloid malignancies through acquisition of loss of function mutations.

**b. Possible oncogenic**

• Previously unreported variants that cluster (±3aa) with known myeloid malignancies related oncogenic variants in COSMIC.

**c. Unknown**

• Variants identified outside the range of frequent variants in genes with known myeloid malignancies related oncogenic variants;

• Variants (even if recurrent) in genes whose role in myeloid malignancies is not yet established.

**Basic sequencing data**

We sequenced 190 genes (Table S2) across 62 patients, resulting in 229

high-confidence mutation. The average gene coverage was 99%. The average read

depth was 540×. Also, 92% of targeted regions were covered with >20×.

By applying the 190-gene panel to the 45 PMF patients, we found 159 mutations in 55

distinct genes with mutation loads varying from 4% to 100% (Table S3). Two (4.4%)

patients with PMF had no mutation. In 17 post-PV/ET MF patients, we found 70

mutations in 29 distinct genes with mutation loads varying from 6% to 93% (Table

S3). All patients with post-PV/ET MF had at least one mutation.

**Analysis of** **clonal architecture**

Variant allele fractions (VAF) can be used to estimate the proportion of tumor cells carrying a given mutation and identify ancestral clonal or subclonal events [3-5]. VAF were calculated as the fraction of mutated reads of the total number of reads of a certain gene. VAFs were adjusted by using copy number information at the locus of each mutation. In our cohort, all loci of gene mutations did not have any copy number changes. We recapitulated the clonal architecture of a patient using these copy number adjusted VAFs. Ancestral vs subclonal events were determined using a copy number-adjusted VAF difference between two events, with a higher VAF indicating ancestral origin. According to the statistically differences in VAF among gene mutations, subjects were classified as two different clonal architecture, namely *ancestral clonal only* and *ancestral clone + sub-clone(s)* (P<0.05).

**Statistical methods**

Correlations between sample groups and clinical and laboratory data were calculated with the χ2 test for qualitative variables with discrete categories and the Mann–Whitney U-test or Kruskal–Wallis analysis of variance for continuous variables.

**References**

1. Li B, Xu J, Wang J, Gale RP, Xu Z, Cui Y, et al. Calreticulin mutations in Chinese with primary myelofibrosis. *Haematologica* 2014; 99 (11): 1697-1700.

2. Papaemmanuil E, Gerstung M, Malcovati L, Tauro S, Gundem G, Van Loo P, et al. Clinical and biological implications of driver mutations in myelodysplastic syndromes. *Blood* 2013; 122: 4616-4627.

3. Nik-Zainal S, Van Loo P, Wedge DC, Alexandrov LB, Greenman CD, Lau KW, et al. The life history of 21 breast cancers. *Cell* 2012; 149: 994-1007.

4. Yates LR, Gerstung M, Knappskog S, Desmedt C, Gundem G, Van Loo P, et al. Subclonal diversification of primary breast cancer revealed by multiregion sequencing. *Nat Med* 2015; 21: 751-759.

5. Molenaar RJ, Thota S, Nagata Y, Patel B, Clemente M, Przychodzen B, et al. Clinical and biological implications of ancestral and non-ancestral IDH1 and IDH2 mutations in myeloid neoplasms. *Leukemia* 2015; 29: 2134-2142.
